# A Novel Preparation-Free Palatal Veneer Technique with Incisal Seating Lugs for Management of Tooth Surface Loss: A Case Report

**DOI:** 10.3390/reports9030269

**Published:** 2026-08-13

**Authors:** Fahad BaHammam

**Affiliations:** 1 College of Dentistry, King Saud bin Abdulaziz University for Health Sciences, Prince Mutib Ibn Abdullah Ibn Abdulaziz Rd, Riyadh 14611, Saudi Arabia; f.bahammam@gmail.com; Tel.: +966-11-4295941; 2 King Abdullah International Medical Research Center, Ahmad Ibn Hanbal Street, Riyadh 11481, Saudi Arabia; 3 Dental Services, Ministry of National Guard—Health Affairs, Prince Mutib Ibn Abdullah Ibn Abdulaziz Rd, Riyadh 14611, Saudi Arabia

**Keywords:** composite resin, palatal veneers, tooth wear, occlusal vertical dimension, case report

## Abstract

**Background and Clinical Significance**: Tooth surface loss is the irreversible loss of dental hard tissue caused by non-carious processes. Restoration of affected teeth can be challenging, especially on the palatal surfaces of maxillary anterior teeth when severe palatal hard-tissue loss is present without marked clinical crown shortening. In such cases, clinicians must preserve the remaining enamel for predictable adhesive bonding while ensuring accurate seating, complete adaptation, and controlled cementation of thin indirect restorations. To address these challenges, this case report describes a novel preparation-free approach in which two temporary incisal seating lugs were incorporated into the design of indirect resin-based composite palatal veneers; **Case Presentation**: A 35-year-old male patient was referred by his general dentist for management of extensive erosive tooth surface loss. Clinical examination revealed generalized tooth surface loss, with a distinctive pattern of severe palatal hard-tissue loss affecting the maxillary anterior teeth without marked clinical crown shortening. Restorative treatment was planned at a 2 mm increase in occlusal vertical dimension. The maxillary anterior teeth were restored with preparation-free indirect resin-based composite palatal veneers, each incorporating two temporary incisal seating lugs to provide a positive seating stop during cementation; the lugs were removed after cementation. Other affected teeth were restored using directly placed resin-based composite restorations. At six months, the veneers remained clinically acceptable, with stable occlusion, maintained pulp sensibility, no postoperative sensitivity, and high patient satisfaction; **Conclusions**: This technique may facilitate restoration positioning during cementation and may represent a useful clinical alternative when seating preparation-free palatal veneers is challenging.

## 1. Introduction and Clinical Significance

Tooth surface loss (tooth wear) is the cumulative, irreversible loss of mineralized dental hard tissue caused by physical and/or chemical processes not related to dental caries (e.g., attrition, abrasion, erosion) [1]. Tooth surface loss is a common condition [2]. Its prevalence increases with age; the proportion of adults with severe tooth surface loss has been estimated to rise from 3% at 20 years to 17% at 70 years [2]. Although control of the etiological factors is the cornerstone of managing tooth surface loss, restoration of the affected teeth may become necessary when function and/or aesthetics have been compromised [3,4]. Restorative treatment may also be indicated to reduce sensitivity associated with dentine exposure, protect the remaining tooth structure, and limit further deterioration of structurally compromised surfaces, particularly when complete control of the etiological factors is uncertain [3,4].

The contemporary restorative management of teeth affected by tooth surface loss has benefited from advances in adhesive dentistry, which have shifted treatment toward additive and minimally invasive restorations [5]. This biologically conservative approach helps avoid further reduction of already compromised teeth while preserving enamel for predictable adhesive bonding [5,6]. However, restoring the palatal surfaces of maxillary anterior teeth in patients with erosive tooth surface loss can still be challenging, particularly in cases characterized by severe palatal hard-tissue loss without marked clinical crown shortening. Direct resin-based composite restorations can be technically demanding when restoring these surfaces, particularly in terms of accurately reproducing the palatal contours intraorally and establishing stable occlusal contacts in maximum intercuspation and harmonious anterior guidance [6]. Indirect palatal veneers, in contrast, allow the palatal anatomy and occlusal scheme to be developed extraorally on articulated casts, providing better control of restoration form, thickness, and occlusal contacts [7].

Although a preparation-free approach is desirable for preserving the remaining enamel and avoiding further reduction of already compromised teeth, the absence of distinct preparation margins, retentive features, or defined seating stops may make accurate orientation, complete seating, and controlled cementation of indirect palatal veneers more difficult when the traditional freehand cementation method is used [3,8]. Several digitally assisted techniques have therefore been proposed to enhance veneer positioning and cementation reliability [8,9,10]. These generally involve guiding devices or indices that either carry and position the veneers on the teeth or are inserted after initial seating to guide, verify, and stabilize the veneers during the cementation process [8,9,10].

Digital veneer workflows have been suggested to improve treatment planning, positioning accuracy, seating stability, and cementation predictability [11]. Nevertheless, their implementation requires additional equipment and software, technical expertise, and financial investment [12]. In addition, their accuracy may be affected by errors introduced during scanning, data alignment, digital design, and manufacturing [12]. Furthermore, the available evidence for digitally guided veneer cementation consists predominantly of case reports and technique papers, with limited quantitative clinical validation [11]. Consequently, a simple positioning method compatible with a conventional workflow remains clinically relevant, particularly where digital guide fabrication is unavailable or impractical [13,14]. To the best of the author’s knowledge, a conventional workflow that facilitates the seating and cementation of preparation-free palatal veneers on anterior teeth without marked clinical crown shortening has not previously been described.

The aim of this case report was therefore to describe the clinical management of a patient with generalized severe tooth surface loss, with particular emphasis on a novel technique used to restore the maxillary anterior teeth, which exhibited extensive palatal hard-tissue loss without marked clinical crown shortening. As part of the overall rehabilitation, the palatal surfaces of these teeth were restored using a conventional preparation-free workflow with indirect resin-based composite veneers incorporating two temporary incisal seating lugs. In the present case, incorporating the seating lugs directly into the veneers enabled the use of a conventional preparation-free workflow without requiring a separately designed and fabricated guiding device or index. The lugs were intended to provide positive seating stops, thereby facilitating restoration orientation and seating during cementation.

## 2. Case Presentation

A 35-year-old male was referred by his general dental practitioner for assessment and management of extensive tooth surface loss and masticatory muscle pain. The patient reported moderate-intensity (approximately 6 out of 10) bilateral dull, aching facial pain and tightness on waking in the morning, which subsided gradually throughout the day. Dietary assessment indicated an intake of approximately two carbonated beverages daily between meals, which were sipped and retained in the mouth for an extended period. Oral hygiene practices included twice-daily toothbrushing with a manual toothbrush and daily interdental cleaning with dental floss. Medical and family histories were unremarkable. Medical consultation and investigation for possible intrinsic acid exposure (e.g., gastro-esophageal reflux disease or eating disorders such as bulimia nervosa) were negative.

Extraoral examination revealed bilateral hypertrophy and tenderness of the masseter and temporalis muscles, supporting a diagnosis of myofascial pain according to the Diagnostic Criteria for Temporomandibular Disorders (DC/TMD). Temporomandibular joint examination was otherwise within normal limits, with no joint tenderness, clicking, crepitus, deviation on opening, or limitation of mandibular movement.

**Figure 1:**
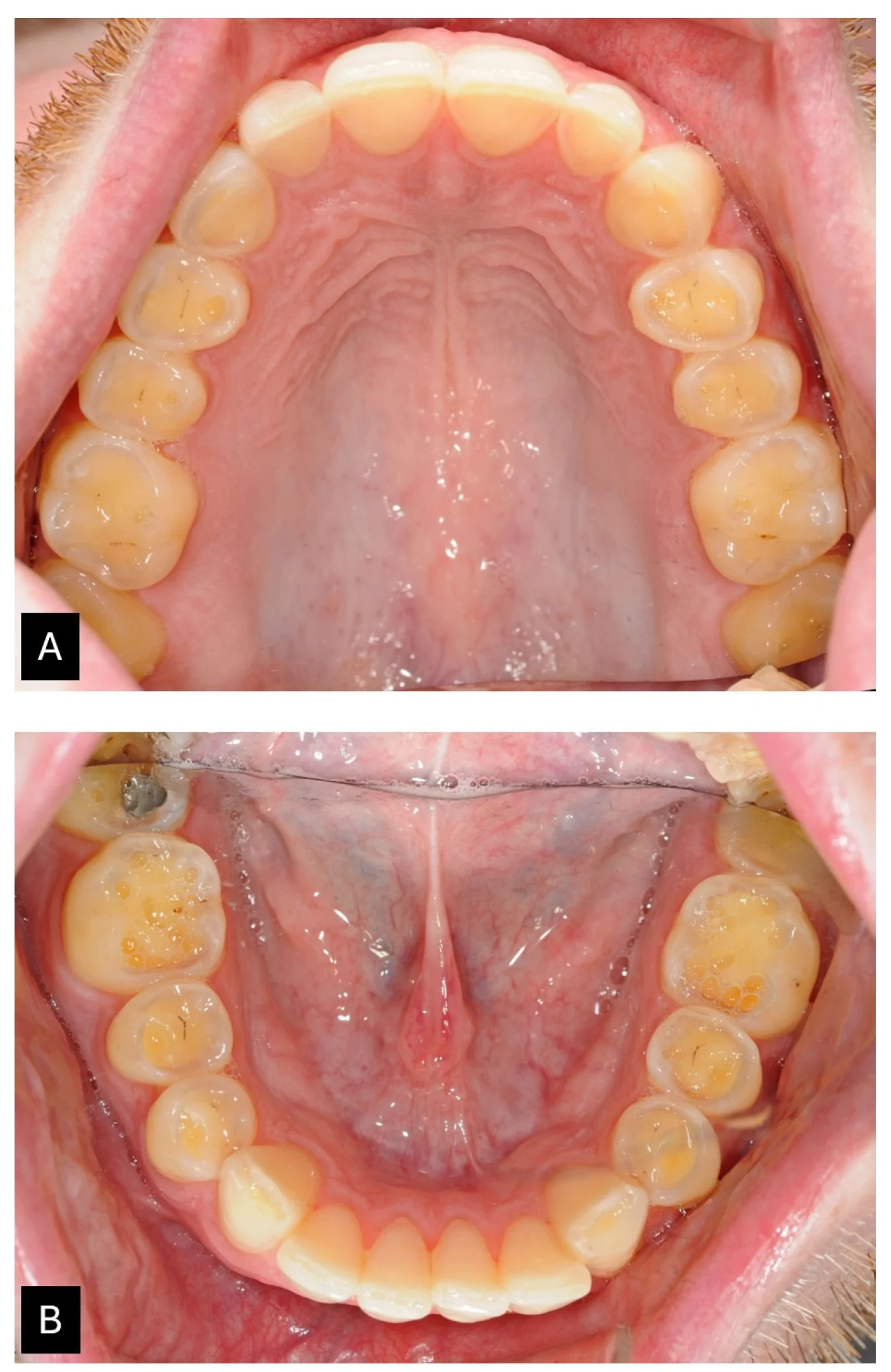
Preoperative occlusal intraoral views of the maxillary (**A**) and mandibular (**B**) arches.

**Figure 2:**
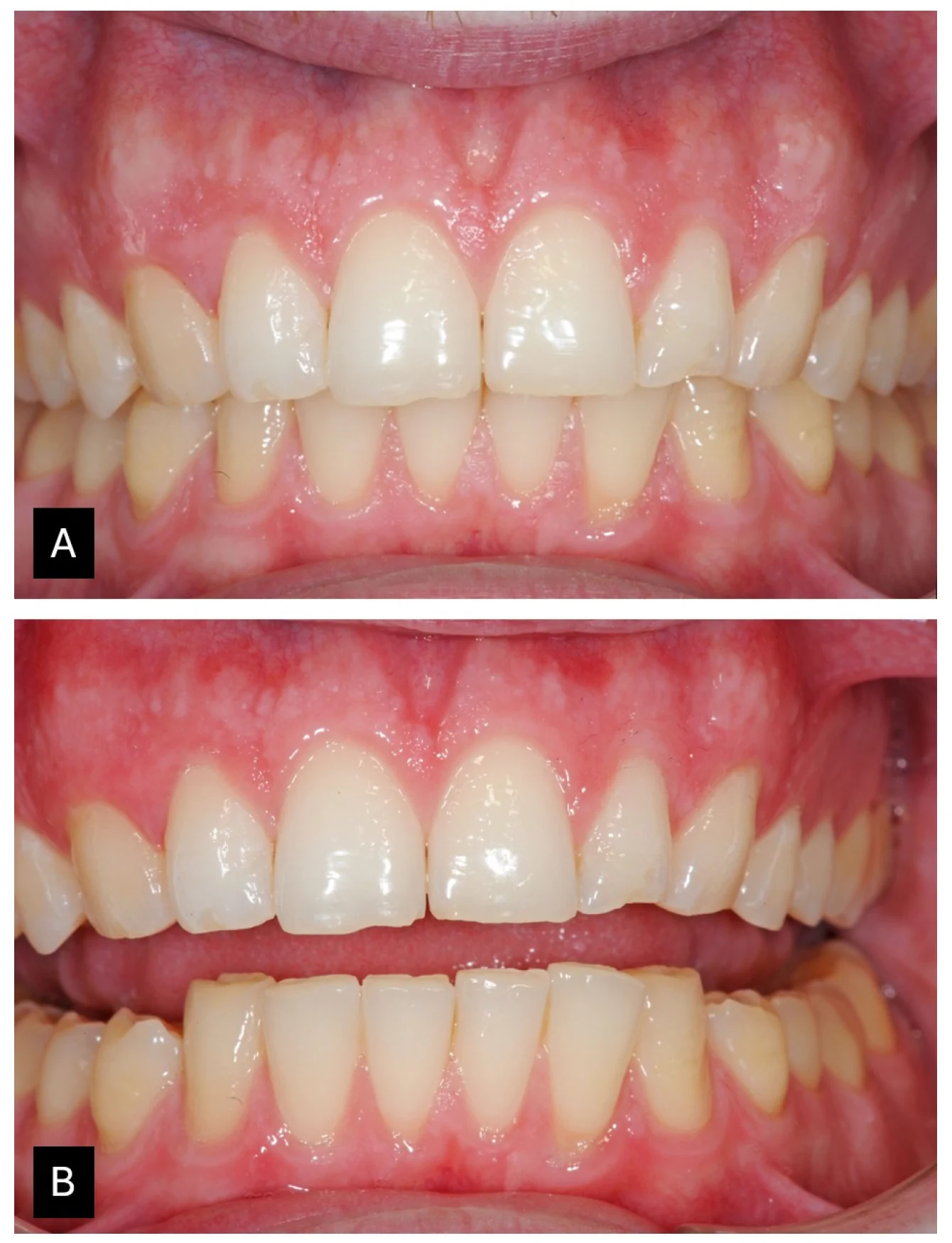
Preoperative frontal intraoral views: maximum intercuspation (**A**) and slightly open mouth view (**B**).

Intraorally, dentition examination revealed generalized tooth surface loss on the occlusal surfaces of the posterior teeth and the palatal surfaces of the maxillary anterior teeth. The severity of tooth surface loss, according to the Smith and Knight Tooth Wear Index, was a score of 4 for the maxillary anterior and mandibular posterior teeth (i.e., tooth surface loss extends to the dentin for more than two-thirds of the tooth’s surface), and a score of 2 for the maxillary posterior teeth and mandibular canines (i.e., tooth surface loss extends to the dentin but for less than one-third of the tooth’s surface) (Figure 1 and Figure 2).

For teeth with a Smith and Knight Tooth Wear Index score of 4, pulp sensibility was assessed using cold testing with ROEKO Endo-Frost cold spray (COLTENE/Whaledent, Altstätten, Switzerland) and electric pulp testing with a Digitest electric pulp tester (Parkell Inc., Edgewood, NY, USA). All tested teeth responded positively, with no findings suggestive of loss of pulp sensibility.

Occlusal examination revealed a bilateral Class II molar and canine relationship in maximum intercuspation. Occlusal contacts in maximum intercuspation and during protrusive and lateral excursions were assessed clinically using double-sided GHM-HANEL Occlusion Foil (12 µm; Hanel-GHM-Medizinal, Nürtingen, Germany). During maximum intercuspation, bilateral, evenly distributed posterior occlusal contacts were present. During lateral excursions, group function occurred on the working side, with no non-working-side interferences. During protrusive excursion, the anterior teeth provided guidance, with disclusion of the posterior teeth. Centric relation was determined using bimanual manipulation, and the initial tooth contact in centric relation (retruded contact position) coincided with maximum intercuspation. The findings of the clinical occlusal examination were confirmed by analysis of diagnostic casts mounted on the semi-adjustable articulator, as described subsequently.

The main clinical events and treatment sequence are summarized in Table 1. The disease-control phase aimed to manage the patient’s myofascial pain and reduce the risk of further tooth surface loss, with suspected sleep bruxism considered a possible contributing factor to both conditions. The patient was advised to perform physical therapy and jaw exercises. The jaw exercises were prescribed to assist in alleviating myofascial pain through gentle stretching of masticatory muscles and controlled, smooth, and symmetrical mandibular movements. In addition, a custom-made, full-coverage mandibular soft occlusal splint was fabricated for use during sleep. The splint was intended for temporary use during the disease-control phase to assist in managing myofascial pain and protect the dentition against further tooth surface loss. The splint was fabricated by vacuum-forming a vinyl sheet over a mandibular cast obtained from an alginate impression. No specific occlusal scheme was incorporated into the splint. It was planned to be replaced with a custom-made maxillary hard acrylic stabilization splint following completion of the restorative phase. To address the potential dietary contribution to tooth surface loss, the patient was also advised to reduce the frequency and amount of carbonated beverage intake; limit it to mealtimes, and to use a straw positioned posteriorly when drinking.

The second phase of the treatment plan aimed to restore the teeth affected by tooth surface loss. Maxillary and mandibular study casts were obtained from alginate impressions. The maxillary cast was mounted on a semi-adjustable Denar^®^ Mark II articulator (Denar Corporation, Anaheim, CA, USA) using a Denar^®^ Slidematic facebow transfer, and the mandibular cast was mounted using a centric relation record made in hard wax following bimanual manipulation. The articulator was programmed bilaterally using values of 25° for sagittal condylar inclination, 15° for progressive side shift, and 0 mm for immediate side shift. A diagnostic wax-up was performed at an increased occlusal vertical dimension. The required increase was determined on the mounted diagnostic casts by opening the articulator incrementally until sufficient interocclusal space was created to provide adequate material thickness for the planned additive restorations without further tooth reduction, while achieving acceptable tooth proportions and aesthetics and permitting the establishment of a stable functional occlusal scheme. A 2 mm increase, measured at the articulator incisal pin, represented the minimum opening required to fulfil these criteria. This increase was incorporated into the diagnostic wax-up and used to guide the subsequent laboratory and clinical procedures. The diagnostic wax-up was designed to establish a mutually protected occlusal scheme, with stable bilateral posterior contacts in maximum intercuspation, anterior guidance with posterior disclusion during protrusive movement, and canine guidance with posterior disclusion during lateral excursions.

An innovative restorative approach was adopted in this case to optimize aesthetics while preserving as much tooth structure as possible, particularly enamel, when restoring teeth affected by tooth surface loss. For the maxillary anterior teeth, preparation-free indirect palatal resin-based composite veneers were planned. A one-step, dual-viscosity polyvinyl siloxane impression of the maxillary arch was taken by simultaneously syringing the light-body Express™ VPS Impression Material around the teeth and seating the tray loaded with the heavy-body Express™ VPS Impression Material (3M, St. Paul, MN, USA). A No. 3 medium upper perforated disposable plastic impression tray, Tray-Aways^®^ (Harry J. Bosworth Company, Skokie, IL, USA), was used after a thin layer of VPS tray adhesive (3M, St. Paul, MN, USA) had been applied to its internal surface.

To transfer the planned maxillomandibular relationship accurately to the maxillary working cast, a sequential cross-mounting procedure was performed. A rigid polyvinyl siloxane interocclusal record (3M™ Imprint™ 4 Bite VPS Bite Registration Material; 3M Deutschland GmbH, Seefeld, Germany) was fabricated bilaterally over the posterior segments between the unaltered posterior teeth of the maxillary diagnostic wax-up cast and the mandibular diagnostic wax-up cast at the planned articulator opening (a 2 mm increase measured at the incisal pin). Using this record, a stone duplicate of the completed mandibular diagnostic wax-up was first cross-mounted against the previously mounted maxillary diagnostic wax-up cast. The maxillary working cast was then cross-mounted against the mounted mandibular duplicate using the same record. During both mounting procedures, the incisal pin was maintained in contact with the incisal table to preserve the planned articulator opening and maxillomandibular relationship. The mandibular duplicate subsequently served as the opposing cast during fabrication and occlusal refinement of the veneers.

**Figure 3:**
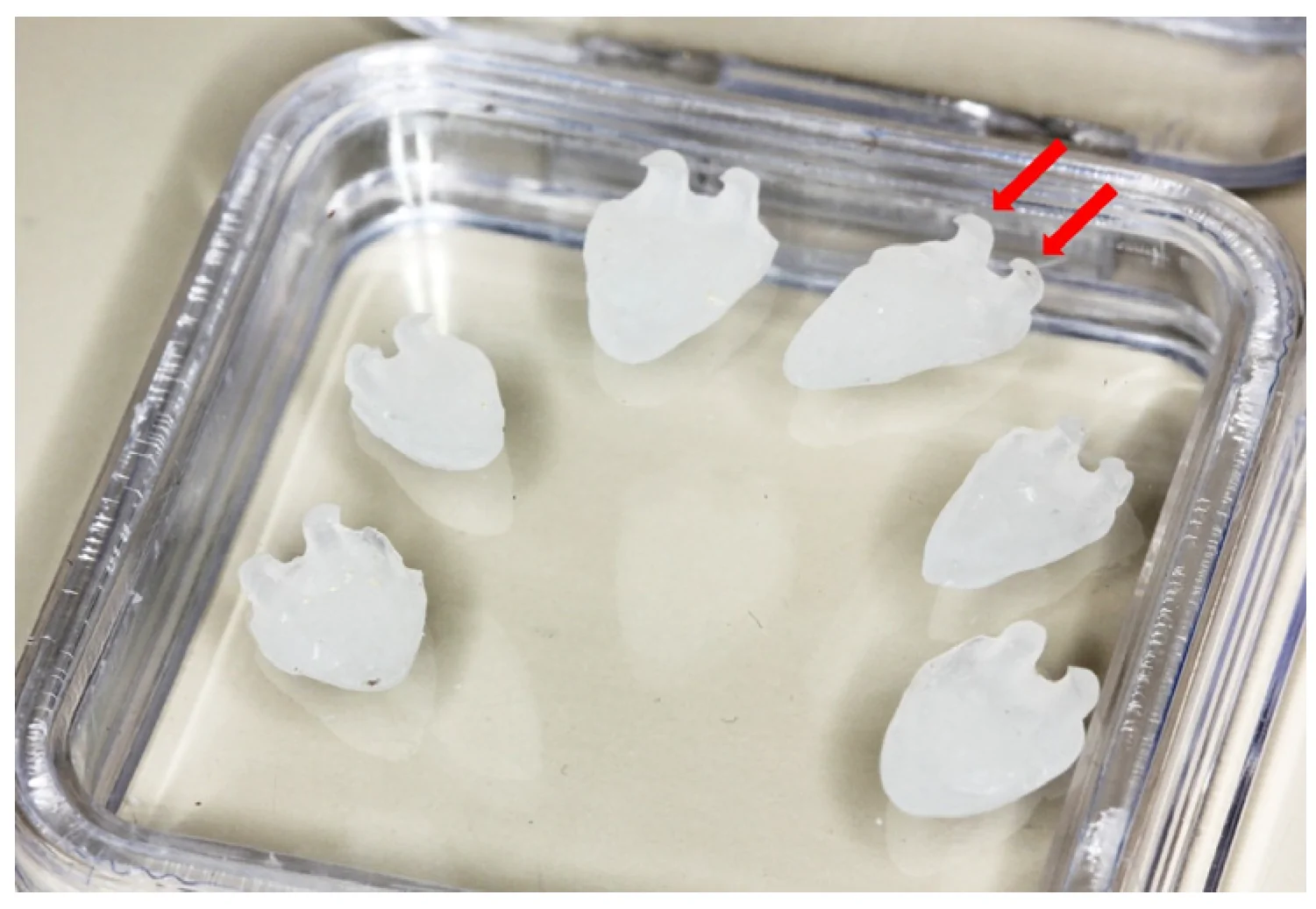
Palatal resin-based composite veneers, each incorporating two temporary incisal seating lugs. The arrows identify the seating lugs on the veneer for the maxillary right central incisor.

The palatal surfaces of the stone dies corresponding to the maxillary anterior teeth were treated with GRADIA^®^ DIE-HARDNER and coated with a thin layer of GRADIA^®^ SEPARATOR (both from GC Europe N.V., Leuven, Belgium). The veneers were fabricated on the dies using the GC GRADIA^®^ Indirect Micro-Ceramic Composite System (GC Europe N.V., Leuven, Belgium). The resin-based composite was applied incrementally directly onto the dies and polymerized in a laboratory light-curing unit in accordance with the material manufacturer’s instructions. To facilitate positioning and complete seating during try-in and cementation, two temporary incisal seating lugs were incorporated into each veneer (Figure 3). Each lug had a rectangular cross-section measuring approximately 1 × 2 mm and was designed to engage the incisal edge and labio-incisal surface of the corresponding tooth, thereby providing a positive seating stop and aiding orientation during try-in and cementation. The palatal morphology and occlusal contacts of the veneers were refined against the opposing mandibular cast to reproduce the occlusal scheme established in the diagnostic wax-up, with the palatal contours designed to provide anterior guidance during protrusive excursion and canine guidance during lateral excursions. The veneers were contoured with Dura-Green^®^ silicon carbide stones, prepolished with CompoMaster^®^ Coarse diamond-impregnated silicone polishers (both from SHOFU Dental GmbH, Ratingen, Germany). Final polishing was performed with GC GRADIA^®^ DIAPOLISHER applied with a felt wheel (GC Europe N.V., Leuven, Belgium).

**Figure 4:**
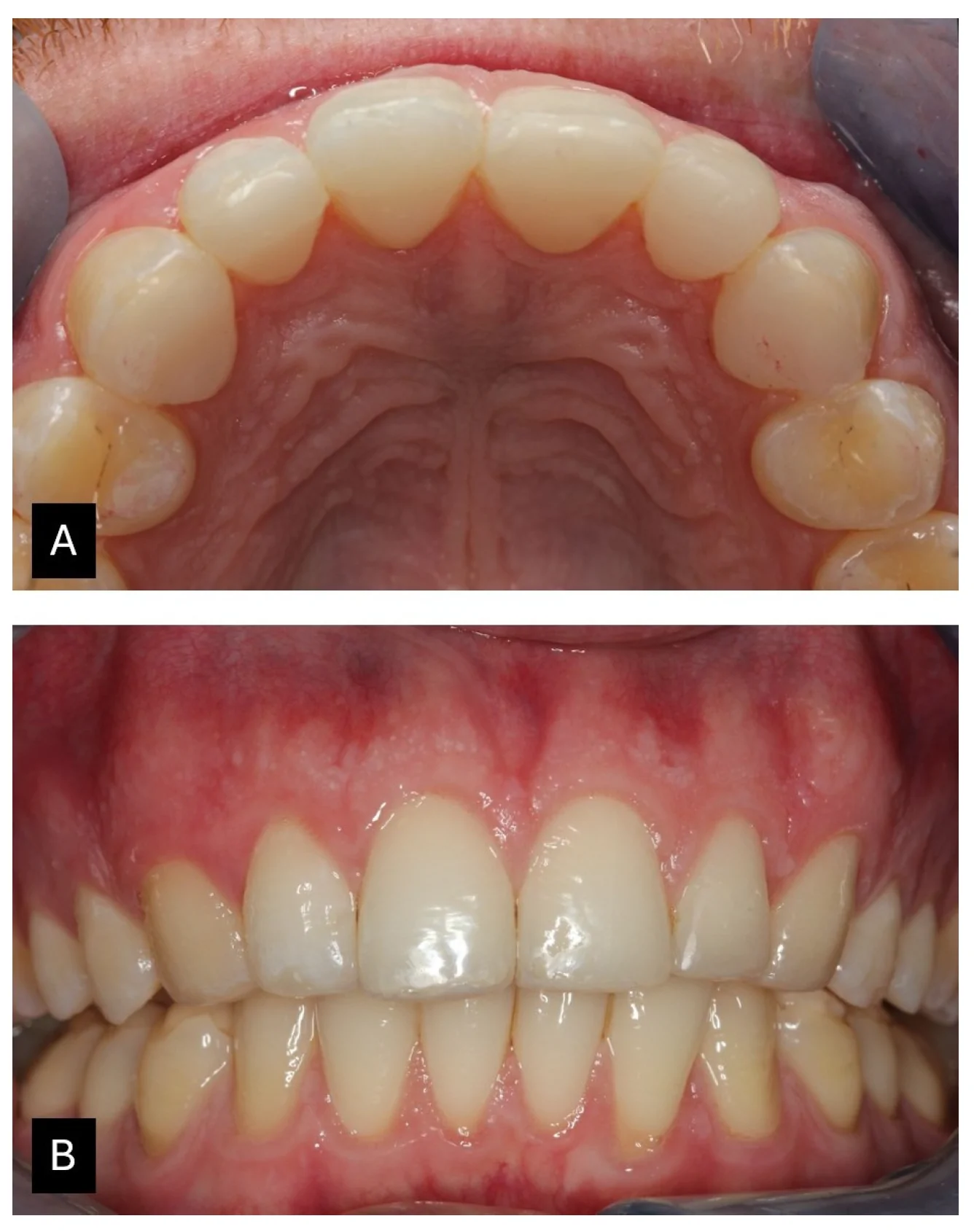
Post-cementation intraoral photographs showing the maxillary occlusal view (**A**) and frontal view in maximum intercuspation (**B**).

During the cementation procedure, the operative field was isolated with cotton rolls, and an OptraGate^®^ retractor (Ivoclar Vivadent AG, Schaan, Liechtenstein) was used to provide soft-tissue retraction and improve access. A gauze throat pack was also placed to reduce the risk of aspiration or ingestion of debris. The palatal surfaces of the maxillary anterior teeth were sandblasted with Korox^®^ 50 (50 µm) (BEGO GmbH & Co., KG, Bremen, Germany), then etched with 37% phosphoric acid for 15 s, thoroughly rinsed, and gently air-dried without desiccating the exposed dentin. OptiBond™ Solo Plus Unidose (Kerr Corporation, Orange, CA, USA) was then applied for 15 s, air-thinned for 3 s, and light-cured for 20 s. The fitting surfaces of the veneers were sandblasted with 3M™ CoJet™ Sand (30 µm) (3M ESPE, Seefeld, Germany), after which ESPE™ Sil Silane Coupling Agent (3M Deutschland GmbH, Neuss, Germany) was applied once and allowed to dry for 5 min. OptiBond™ Solo Plus Unidose was subsequently applied to the fitting surfaces. The veneers were then seated using RelyX™ Veneer Cement (3M, St. Paul, MN, USA), and the excess cement was removed before final light polymerization, which was performed for 30 s from each accessible direction. The temporary incisal seating lugs were subsequently removed using a finishing diamond bur under water cooling while high-volume suction was maintained immediately adjacent to the operative site. Any visible fragments were retrieved, and the oral cavity was inspected before the gauze throat pack was carefully removed. This was followed by finishing and polishing of the incisal areas (Figure 4).

**Figure 5:**
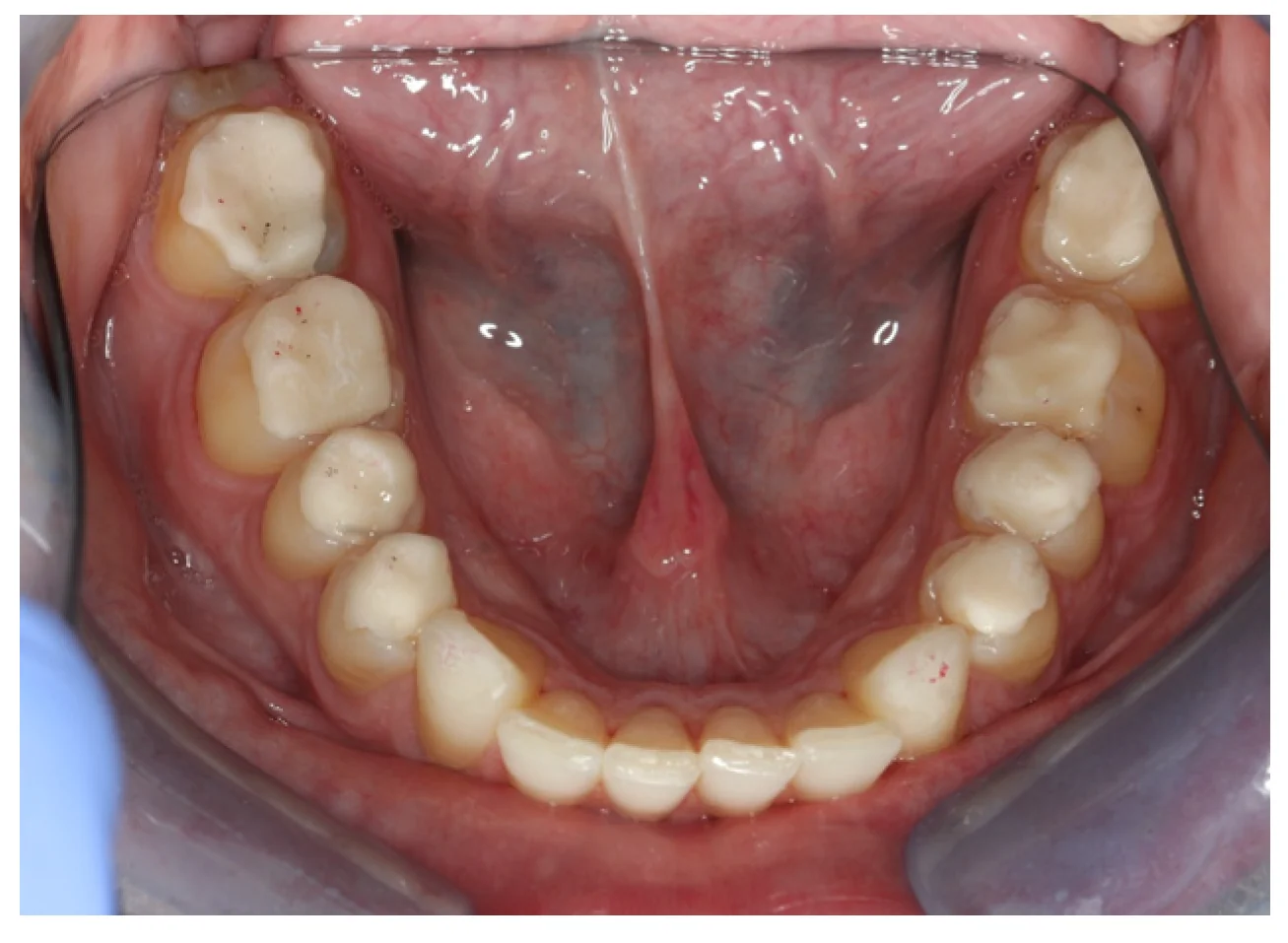
Mandibular occlusal view showing high-viscosity glass ionomer temporary restorations placed on the posterior teeth to preserve the interocclusal space.

At the same appointment, provisional restorations were placed on the mandibular canines and all mandibular posterior teeth using a high-viscosity glass ionomer cement (Fuji IX GP Extra, GC, Japan) to maintain the interocclusal space created by the planned increase in occlusal vertical dimension for the definitive posterior restorations (Figure 5). Before placement of the provisional restorations, the tooth surfaces were conditioned with GC DENTIN CONDITIONER (GC Corporation, Tokyo, Japan) for 20 s, thoroughly rinsed, and gently air-dried without desiccation. The high-viscosity glass ionomer cement was selected as a material of choice to permit efficient restoration of the 10 mandibular teeth during the same visit, given its less technique-sensitive placement protocol compared with definitive resin-based composite restorations.

Following cementation of the veneers and placement of the provisional restorations, interproximal contacts were assessed using dental floss. Static and dynamic occlusal contacts were evaluated intraorally using double-sided 12 μm GHM-HANEL Occlusion Foil. Static contacts were adjusted to achieve even bilateral posterior contacts in maximum intercuspation, whereas dynamic contacts were adjusted to provide smooth anterior guidance during protrusion and canine guidance during lateral movements, with posterior disclusion. Together, these static and dynamic relationships established a mutually protected occlusal scheme. The temporary restorations were later replaced with nanohybrid composite resin restorations (Filtek™ Supreme XTE, 3M, United States).

Following completion of the restorative phase, the provisional mandibular soft splint was replaced with a new maxillary hard stabilization splint. New alginate impressions of the maxillary and mandibular arches were obtained, and the resulting casts were mounted on an articulator in centric relation using a hard-wax interocclusal record obtained following bimanual manipulation, as described above. A full-coverage maxillary stabilization splint was then fabricated from heat-polymerized acrylic resin. Occlusal contacts and guidance were adjusted clinically using double-sided 12 μm GHM-HANEL Occlusion Foil and HANEL Shimstock-Foil (8 μm; COLTENE, Germany) to establish a mutually protected occlusal scheme, with evenly distributed bilateral contacts in centric relation and smooth, shallow anterior guidance during excursive movements. The patient was instructed to wear the splint during sleep to assist in managing temporomandibular disorder symptoms and protecting the recently placed restorations. At the one-week follow-up, the splint and its occlusal contacts and guidance were reassessed, and further adjustments were made as necessary.

**Figure 6:**
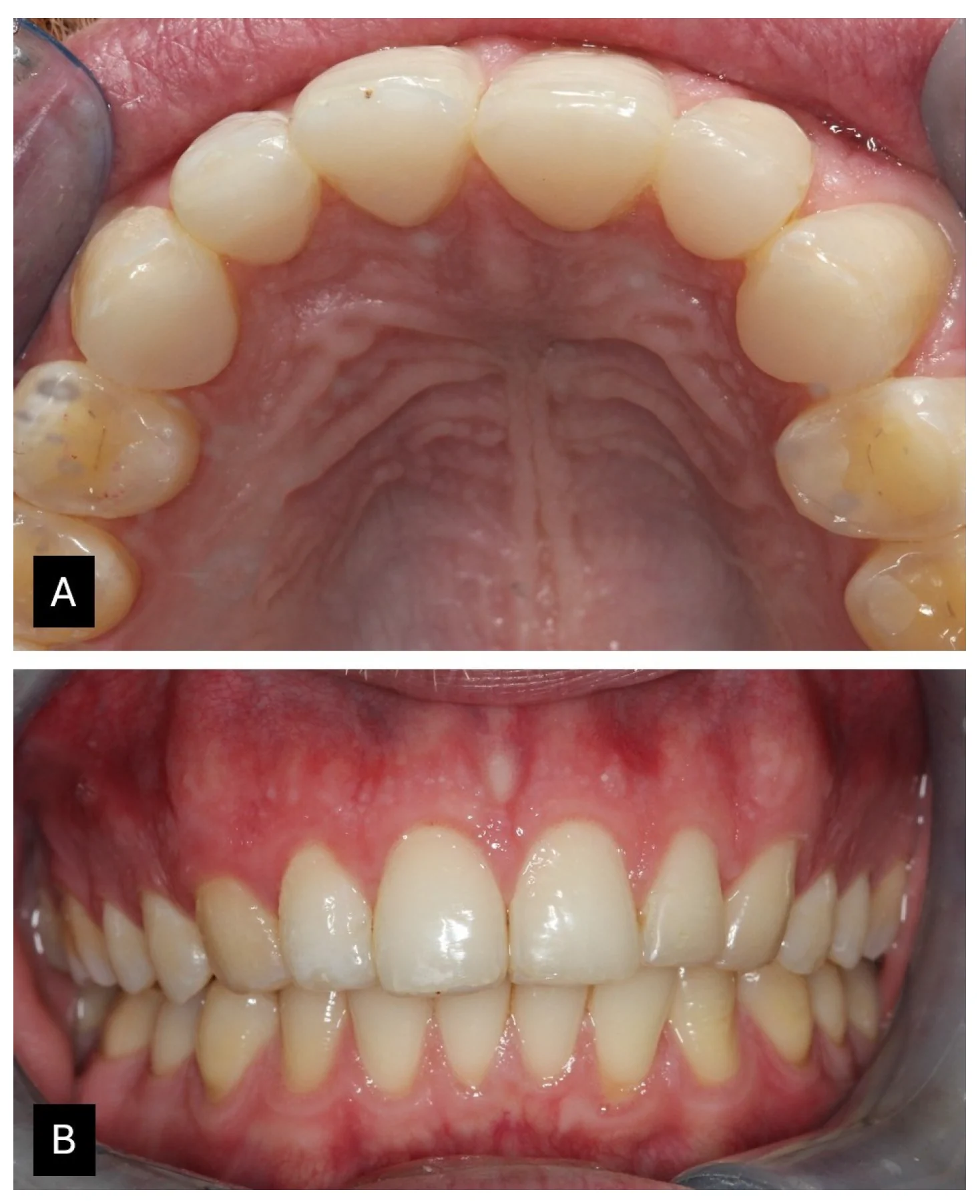
Six-month follow-up intraoral photographs showing the maxillary occlusal view (**A**) and the frontal view in maximum intercuspation (**B**).

At the six-month follow-up, the patient reported resolution of the bilateral dull, aching facial pain and tightness usually experienced upon waking in the morning. Extraoral examination revealed that, although bilateral hypertrophy of the masseter and temporalis muscles persisted, muscle tenderness had resolved. Other findings of the temporomandibular joint examination were within normal limits. Occlusal stability was assessed clinically using double-sided 12 μm GHM-HANEL Occlusion Foil at maximum intercuspation and during protrusive and lateral excursions. The examination confirmed maintenance of the planned mutually protected occlusal scheme, including even bilateral posterior contacts in maximum intercuspation, smooth anterior guidance during protrusion, canine guidance during lateral movements, and posterior disclusion, with no detectable changes from the occlusal relationships established upon completion of the restorative phase. The patient reported comfortable mandibular function, with no difficulty in mastication or speech. Collectively, the clinical and patient-reported findings indicated good adaptation to the increase in occlusal vertical dimension. The palatal veneers remained clinically acceptable, with no evidence of debonding, or marginal or surface deterioration (Figure 6). Pulp sensibility was maintained, as confirmed by cold and electric pulp testing performed as described above. The patient also reported no postoperative sensitivity. Overall, the patient was satisfied with the aesthetics and function of the restorations. The prognosis was considered favorable, provided that the patient continued dietary control, occlusal splint use, and regular maintenance.

## 3. Discussion

This case illustrates a distinctive, likely multifactorial pattern of tooth surface loss associated with dietary acid exposure and possible mechanical wear, to which suspected sleep bruxism may have contributed. The distribution and morphology of the surface loss suggest that the pattern was most consistent with erosion as a major contributing factor, most evident in the extensive palatal loss affecting the maxillary anterior teeth, with minimal reduction in clinical crown length. Further support for an erosive component is provided by the characteristic “cupping” of the posterior occlusal surfaces, a feature commonly associated with acid-related erosion [15]. Although a similar presentation may be seen with intrinsic acid exposure (e.g., gastro-esophageal reflux disease or eating disorders such as bulimia nervosa), medical consultation excluded these causes in this patient.

The first phase of the patient’s treatment plan aimed to control modifiable risk factors contributing to ongoing tooth surface loss. Adopting this cost-effective approach as the first line of treatment is crucial to ensure a favorable long-term prognosis. A tailored dietary counseling intervention, incorporating behavior change techniques, was adopted, as this approach has been shown to be more effective than standard dietary advice in reducing dietary acid intake and, subsequently, the rate of erosive tooth surface loss [16,17]. Dietary counseling was supplemented by prescribing a temporary soft splint to be worn during sleep, which was subsequently replaced with a hard stabilization splint following completion of the restorative phase. Although both splints were primarily prescribed to help control myofascial pain [18], clinical evidence suggests that occlusal splints may also reduce the rate of tooth surface loss [19]. A hard acrylic splint was selected for long-term use because of its greater wear resistance and durability compared with the vacuum-formed splint [20]. These properties were considered particularly advantageous because suspected sleep bruxism was considered a possible contributing factor [20].

The definitive phase of the treatment plan aimed to protect the structural integrity of the affected teeth, establish a stable occlusion, and cover exposed dentine surfaces, which are at higher risk of accelerated tooth surface loss [21]. To achieve these objectives, an ultra-conservative, preparation-free restorative approach was adopted. This approach prioritized maximal preservation of remaining tooth structure, particularly enamel. Enamel provides more reliable and durable adhesion than dentine, thereby improving the predictability and longevity of bonded restorations [22]. In addition, avoiding further tooth reduction helps protect the pulp, as teeth affected by tooth surface loss often have reduced residual dentine thickness; additional preparation may increase the risk of pulpal irritation or exposure and associated complications [23]. Finally, unnecessary preparation may further weaken an already compromised tooth, increasing the risk of biomechanical failure [24].

Restoring the palatal surfaces of the maxillary teeth while adopting this ultra-conservative, preparation-free restorative approach can be challenging due to the lack of a geometric lock that guides the palatal veneers into a single, stable terminal position during cementation. To overcome this challenge, two temporary incisal seating lugs were incorporated into the design of the veneers. The concept of an incisal seating lug was originally developed for resin-bonded fixed partial dentures as a supplementary extension of the metal framework [25]. It is often designed as a small hook, approximately 1.0–1.5 mm in width and 0.5–1.0 mm in thickness, projecting 1–2 mm from the lingual aspect, to engage a specific anatomical stop on the abutment tooth, typically the incisal edge or occlusal surface [25].

The main purpose of the incisal seating lug is to provide a definite vertical stop and a positive index for orientation during the cementation phase [25]. In this case, they were also intended to provide stable handling points and facilitate the application of controlled pressure along the seating path. Theoretically, this may help overcome the hydraulic pressure generated by the viscous luting cement and facilitate seating of the indirect restoration onto the tooth surface [26]. This can also theoretically help minimize the luting cement film thickness, which has been associated with improved bond strength and the clinical success of indirect restorations [27]. Even though seating lugs are often severed and polished after cementation to optimize aesthetics and minimize occlusal interference, some clinicians may retain these incisal extensions, particularly in resin-bonded fixed partial denture cases, when they are intentionally designed to function as definitive rests to enhance resistance to displacement [25].

A potential advantage of this technique is that it addresses a specific clinical challenge encountered when cementing preparation-free palatal veneers, namely, the absence of conventional preparation features to guide seating and orientation. Compared with freehand cementation, the seating lugs may provide visible orientation references and positive seating stops. Accordingly, the technique may aid restoration positioning during try-in and cementation while preserving the preparation-free nature of the restorations. Unlike separate positioning indices or guiding devices, the lugs are incorporated directly into the restorations, thereby eliminating the need to fabricate and manipulate an additional device during cementation while potentially allowing better access for excess-cement removal and light polymerization [8,9,10]. However, the seating lugs require an additional laboratory step and must be carefully removed after cementation, with subsequent finishing and polishing of the incisal surfaces. During lug removal, there is a risk of ingestion or aspiration of detached fragments or debris; therefore, a gauze throat pack should be used to reduce this risk [25]. Another potential complication is incomplete removal of the lugs, which may leave residual irregularities that can act as plaque-retentive sites [25].

Several limitations, however, should be considered when interpreting the findings of this case report. As a single-patient report, this case cannot establish the clinical superiority of the proposed technique over freehand or guided cementation techniques. The report is also limited by a short six-month follow-up. Although favorable clinical outcomes were observed during this period, the limited follow-up does not permit reliable evaluation of the long-term durability, wear resistance, marginal integrity, or prognosis of the preparation-free palatal veneers. In addition, seating accuracy and luting cement film thickness were not quantitatively measured; therefore, this report cannot establish whether the lugs improved seating accuracy or minimized the luting cement film thickness. Another limitation is that treatment outcomes were not assessed using objective quantitative measures. For example, although occlusal stability was assessed clinically using occlusal foil, no computerized or other quantitative analysis of occlusal contact timing or force distribution was performed. Last, future integration of AI-assisted tools may enhance diagnosis, treatment planning, and follow-up, thereby complementing the conventional restorative workflow described in this report.

Future research should include laboratory studies comparing the proposed technique with freehand cementation and digitally designed positioning guides and indices in terms of seating accuracy and cement-film thickness. Prospective clinical studies with larger samples and longer follow-up are also needed to evaluate outcomes such as wear, marginal adaptation, discoloration, fracture, debonding, and long-term occlusal stability. Until such evidence is available, the findings of this single case should be considered preliminary.

## 4. Conclusions

This case describes the comprehensive rehabilitation of a patient with generalized severe tooth surface loss, including a distinctive pattern characterized by extensive palatal hard-tissue loss of the maxillary anterior teeth without a marked reduction in clinical crown height. This pattern complicated accurate seating of the preparation-free palatal veneers. Two temporary incisal seating lugs were incorporated into each veneer to address this challenge, provide positive seating stops, and facilitate positioning. At the six-month follow-up, the restorations remained clinically acceptable, with high patient satisfaction. Within the limitations of this single case report using standard data, this case highlights the importance of assessing patient-specific occlusal determinants and using the findings to guide individualized occlusal reconstruction in individuals with generalized pathological tooth surface loss. Additionally, the presented technique for seating preparation-free palatal veneers may facilitate restoration positioning during cementation and may represent a useful clinical alternative when accurate seating is challenging.

## Figures and Tables

**Table 1:** Timeline of the episode of care.

Stage	Clinical Event
Initial referral	Referred for management of extensive tooth surface loss and masticatory muscle pain.
Diagnostic phase	Dietary assessment, extraoral and intraoral examination, tooth wear scoring, pulp sensibility testing, occlusal assessment, and medical consultation to exclude intrinsic acid exposure.
Disease-control phase	Dietary counseling, physical therapy and jaw exercises, provision of a lower occlusal soft splint.
Restorative planning	Mounted study casts, diagnostic wax-up, and planned 2 mm increase in occlusal vertical dimension.
Restorative phase	Preparation-free indirect palatal resin-based composite veneers with temporary incisal seating lugs were cemented. Temporary high-viscosity glass ionomer cement restorations were placed to maintain the interocclusal space and later replaced with nanohybrid composite resin restorations.
Six-month follow-up	Veneers clinically acceptable, with no debonding or marginal/surface deterioration; occlusion stable; pulp sensibility maintained; no postoperative sensitivity; patient satisfied with aesthetics and function.

## Data Availability

The original contributions presented in this study are included in the article. Further inquiries can be directed to the corresponding author.
